# Editorial: The evolving role of liver transplantation for the treatment of malignant tumors: current perspectives and future directions

**DOI:** 10.3389/fsurg.2025.1721613

**Published:** 2025-10-29

**Authors:** Derek A. DuBay, Altan Alim, Roberto Gedaly

**Affiliations:** 1 Department of Surgery, Prisma Health, Greenville, United States; 2 Department of Surgery, University of Kentucky, Lexington, United States

**Keywords:** liver transplantation, liver transplantation - outcome, Milan Criteria (MC), beyond Milan Criteria, neuroendocrine liver metastases, metastatic colorectal cancer (mCRC), sarcopenia in cancer patients

The oncologic indications for liver transplantation continue to evolve with increasing sophistication and expanding clinical relevance. In this special issue of *Frontiers in Surgery*, we present a Research Topic of five articles that collectively provide an in-depth overview of the current landscape of liver transplant oncology. The contributing authors—representing centers across North America, Europe, and Asia—offer diverse yet complementary perspectives that capture the dynamic evolution of this rapidly advancing field. Together, these contributions highlight a pivotal trend: the ongoing refinement and expansion of transplant criteria for hepatocellular carcinoma (HCC) and selected metastatic malignancies. Each article critically examines seminal studies that have shaped modern transplant oncology, while also exploring the increasingly nuanced process of patient selection in the era of new systemic therapy options and traditional surgical resection approaches.

## The role of radiological interventions in hepatocellular carcinoma before liver transplantation: a surgical perspective

Hepatocellular carcinoma (HCC) remains the most frequent oncologic indication for liver transplantation after end-stage liver disease. In this insightful manuscript, Alim and colleagues provide a comprehensive review the evolving role of radiological interventions as both bridging and downstaging strategies in liver transplant candidates. The authors synthesize current evidence on the use of locoregional therapies—including transarterial chemoembolization (TACE), transarterial radioembolization (TARE), radiofrequency ablation (RFA), and microwave ablation (MWA)—to optimize patient selection and timing for liver transplantation. Importantly, they emphasize that these modalities not only maintain disease control and reduce waiting list dropout but also function as a biological “stress test” that helps identify tumors with indolent behavior while excluding those with aggressive phenotypes. By integrating oncologic response, radiologic findings, and surgical feasibility, the review underscores the growing consensus that locoregional therapies serve not merely as bridging treatment, but as valuable diagnostic and prognostic tools that refine transplant eligibility and ultimately improve posttransplant outcomes.

## Liver transplantation for HCC within and beyond Milan Criteria: single-center experience with literature review

Tırnova and Kanmaz revisit one of the most fundamental and enduring questions in transplant oncology: how far can we safely extend the selection boundaries for hepatocellular carcinoma (HCC) beyond the Milan Criteria? This manuscript offers an insightful and concise overview of the ten proposed alternative extended HCC criteria, providing a global perspective on the debate. The authors meticulously review the comparative data between the Milan criteria vs. these extended criteria, underscoring the growing importance of tumor biology in transplant candidacy. Critical pre-transplant surrogates of tumor biology—such as alpha-fetoprotein (AFP) levels, tumor differentiation, total tumor diameter, and the response to bridging therapies—are increasingly recognized as essential to the patient selection process. By contextualizing these findings within contemporary literature and describing their own experience incorporating these extended criteria into their transplant program, this article highlights the global convergence toward biologically driven rather than morphologically restricted transplant eligibility for HCC.

## Liver transplantation for the treatment of neuroendocrine liver metastases

Orozco et al. present a comprehensive summary of the current data surrounding the controversial indication of liver transplantation for neuroendocrine (NE) liver metastases (NELM). Drawing upon data from major international guidelines—including Milan-NET, OPTN/UNOS, and ESMO—the authors delineate the complex criteria for optimal candidate selection. They emphasize that long-term survival is achievable in carefully chosen patients with well-differentiated, low-grade tumors, limited hepatic involvement, and absence of extrahepatic disease. Importantly, this review highlights that liver transplantation, while controversial, can provide excellent outcomes when applied within strict biological and anatomical boundaries. This work serves as an important contribution to a broader discussion on the viability of liver transplantation as a treatment option for metastatic cancers.

## The evolving role of liver transplantation for metastatic colorectal cancer: current perspectives and future directives

Bendersky et al. provide a detailed and timely review of liver transplantation for metastatic colorectal cancer (mCRC), one of the most debated frontiers in transplant oncology. Drawing on pivotal trials such as SECA-I, SECA-II, and the recent TransMet study, the authors trace the evolution of patient selection and outcomes that have reshaped this controversial indication. They highlight the prognostic value of the Oslo scoring system and the influence of genetic profiling—particularly BRAF and RAS mutations—on post-transplant survival. Moreover, their discussion of MELD exception policies and advances in donor utilization via normothermic machine perfusion underscores how innovation in allocation and technology is redefining the feasibility of transplantation for select mCRC patients.

## Impact of low preoperative appendicular skeletal muscle mass on postoperative complications and short-term outcomes in liver transplant recipients

The final article in this series addresses an often overlooked but critically important issue: the relationship between sarcopenia and post-liver transplant outcomes. Xu et al. from West China Hospital present a well-designed, propensity score–matched study on behalf of the Asian Working Group for Sarcopenia examining the influence of sarcopenia on postoperative outcomes in liver transplant recipients. Their findings reinforce the growing body of evidence that links sarcopenia, a finding quite common in oncology patients, to increased morbidity and mortality in liver transplant recipients. This article provides essential insights into the multifaceted implications of sarcopenia for patient care, underscoring the importance of addressing muscle wasting as part of the comprehensive management of patients undergoing liver transplantation.

## Conclusion

Collectively, the five articles in this special edition reflect the remarkable transformation of liver transplantation in the oncologic and multidisciplinary context. Once constrained by rigid criteria, the field is now propelled by biological insight, technological innovation, and refined patient selection. From radiologic downstaging to transplanting select patients with metastatic disease, and from understanding sarcopenia to redefining prehabilitation, each contribution underscores the growing sophistication of transplant oncology. As these advances continue to shape practice worldwide, liver transplantation stands poised to remain not only a curative option for advanced liver disease but also a beacon of progress in surgical oncology.

